# Evaluating outcomes of the child and adolescent psychiatric unit: A prospective study

**DOI:** 10.1186/1752-4458-5-7

**Published:** 2011-03-31

**Authors:** Yutaro Setoya, Kazuhiko Saito, Mari Kasahara, Kyota Watanabe, Masaki Kodaira, Masahide Usami

**Affiliations:** 1 National Institute of Mental Health, National Center of Neurology and Psychiatry, 4-1-1 Ogawa-Higashi, Kodaira, Tokyo, 187-8553, Japan; 2 Kohnodai Hospital, National Center for Global Health and Medicine, Japan; 3 National Center for Child Health and Development, Japan

## Abstract

**Background:**

The aims of this prospective study are to clarify the outcomes of child psychiatric inpatient treatment and to identify factors associated with patient improvement.

**Methods:**

The attending psychiatrist used the Children's Global Assessment Scale (CGAS) to assess youths at admission to and discharge from a child and adolescent psychiatric unit in Japan(N = 126, mean age = 12.8, SD = 1.9). Hospital records gathered sociodemographic and clinical variables. In addition, youths and their primary caregivers assessed themselves using the Youth Self Report (YSR) and the Child Behavior Checklist (CBCL), respectively. Longitudinal analyses compared each scales' baseline and discharge scores. We also examined factors associated with changes in functioning (CGAS).

**Results:**

Longitudinal comparisons revealed that CGAS, CBCL and YSR scores showed improvement over time (CGAS: t = -14.40, p = 0.00; CBCL: t = 3.80, p = 0.00; YSR: t = 2.40, p = 0.02). Linear regressions determined that the factors associated with improvement in CGAS included age, lower CGAS scores at admission, frequency of group therapy and psychiatric diagnosis.

**Conclusions:**

This evaluation of children and adolescents in an inpatient unit demonstrated clinical improvement over time and identified factors associated with said improvement.

## Background

In Japan, youth mental health is a serious issue. Press reports are increasing regarding youth problem behaviors such as sensational crimes, increases in school refusal and social withdrawal, group suicides and self-harming behaviors. Children with psychiatric disorders are responsible for some, but not all, problematic behaviors in society. In a review of epidemiological studies, Roberts et al. reported that as many as one in five children and adolescents have a diagnosable mental disorder [1]. There are no empirical data of the prevalence of psychiatric disorder in child and adolescent in Japan. The national survey estimates, however, that the ratio of child with diagnosable mental disorder seen at outpatient setting has more than doubled in 12 years (from 85 per 10,000 children under 15 years old in 1996 to 182 in 2008) [2].

Among current mental disorder interventions for children, inpatient treatment is the most restrictive and invasive. Inpatient treatment provides opportunities for intensive intervention but risks significantly disrupting the child's life and is expensive. Knowing whether children benefit from inpatient care is therefore important, and evaluating child psychiatric inpatient unit outcomes is critical [3]. Moreover, determining which aspects of inpatient treatment are most helpful to clients may help improve the organization of service [4]. In their review of child psychiatric inpatient treatment, Blantz et al. concluded that little is known about inpatient treatment, including factors that influence hospital admission, content of care in the hospital, the inpatient arrangements that result in the best outcomes and the connections with necessary aftercare services [5]. In addition, there have been calls for the inclusion of assessment from multiple observers [6]. Recently, some studies were conducted reflecting methodological improvements of this nature [4,7].

In Japan, child and adolescent psychiatric inpatient services are still scarce, and only less than 20 hospitals have a child and adolescent psychiatric inpatient unit. For the population of Japan (128 million), this is clearly insufficient, and many children suffering psychiatric disorders are admitted to ordinary psychiatric inpatient units, with adults [8].

In terms of service research, no empirical study of Japanese child psychiatric inpatient treatment has been performed.

The aims of this prospective study are to clarify the outcomes of child psychiatric inpatient treatment and to identify the factors associated with improvement.

## Methods

### Hospital setting

We recruited participants from the child and adolescent psychiatric unit of Kohnodai Hospital, National Center for Global Health and Medicine, a leading Japanese hospital for the treatment of children with psychiatric problems; it is also one of the few hospitals that accept medical residents for child and adolescent psychiatry. In fact, many child psychiatrists working in other child and adolescent units had their residency here.

In this open unit, a clinical team consisting of child psychiatrists, nurses, psychologists, and social workers evaluates children and arranges for their treatment. The unit has 41 beds divided across nine single rooms and eight four-person rooms. Targeted patients for this unit are under 15 years old. Lengths of stay vary, from as short as a few days to as long as five years.

All cases of inpatient treatment consisted of Milieu Therapy, nurse care, individual psychotherapy, and parent guidance or family therapy. Psychopharmacology is prescribed for a majority of the cases. In addition, occupational therapy and/or group therapy sessions are offered biweekly. Occasional excursions under hospital auspices are conducted with the permission of the attending psychiatrist. There is an in-hospital school up to 9^th ^grade, for longer-stay patients. Also, there are monthly family groups which families can join freely and talk about their concerns with other participants and facilitating psychiatrist and nurses in a secured environment.

### Participants

Patients admitted to the child and adolescent unit of Kohnodai hospital between October 1, 2002 and March 31, 2005, as well as 32 additional children already in the unit participated in this study. Three patients were not discharged on March 31, 2006 and were excluded from analyses. Written informed consent was obtained from all the participants and the parents for obtaining data and for publication of this report.

### Assessment questionnaire

The attending psychiatrist assessed all participants. The assessment included the DSM-IV and the Children's Global Assessment Scale (CGAS) [9,10]. In Japan, both DSM-IV and ICD-10 are widely used by psychiatrist, and we re-diagnosed the children at admission and discharge using DSM-IV. If the diagnosis were different between admission and discharge, diagnosis at discharge were used. In addition, the psychiatrist, researcher (Y.S.), or both recorded sociodemographic and clinical information (i.e., age, sex, level of intelligence, aim of admission, past admission experience, family structure, presence of self-harming or disruptive behavior and treatment during the stay).

In addition, the child's primary caregiver and the children themselves were asked to complete the Child Behavior Checklist (CBCL) and the Youth Self Report (YSR), respectively [11,12].

### Scales

#### Children's Global Assessment Scale (CGAS)

The CGAS is an adaptation of the Global Assessment Scale (GAS) designed to reflect the lowest level of functioning for a child or adolescent during a specified time period [10]. As with the GAS, its values range from 1 to 100. Scores above 70 indicate normal functioning. The Japanese version of the CGAS has good validity [13,14].

#### Child Behavior Checklist (CBCL)/Youth Self Report (YSR)

The CBCL and YSR are designed for use by caregivers and children, respectively, to provide standardized reports of children's adaptive functioning as well as recent emotional and behavioral problems [11,12]. Problematic behaviors are scored in terms of eight syndromes (withdrawn, somatic complaints, anxious/depressed, social problems, thought problems, attention problems, delinquent behavior, and aggressive behavior) and three scales (internalizing, externalizing and total Problems). The Japanese versions of CBCL and YSR have good reliability and validity [15,16].

### Statistical Analyses

To assess the effectiveness of inpatient treatment and identify the characteristics of children and families who benefit most from inpatient treatment, a paired t-test compared scores on each scale at baseline and discharge.

Pearson and Spearman correlation coefficients measured the improvement in CGAS scored between baseline and discharge for scale scores; t-tests or ANOVAs did the same for categorical factors.

We conducted a linear regression with CGAS improvement as the dependent variable to further clarify the importance of various factors.

All of the analyses were conducted using SPSS 15.0J for Windows.

### Collection rate of questionnaires

Collection rate for attending psychiatrist's assessment and clinical characteristic for baseline and discharge were both 100%. At baseline, we collected 63.5% (n = 80) questionnaire from caregivers and 51.6% (n = 65) from children; at discharge, these rates decreased to 54.0% (n = 68) and 47.6% (n = 60), respectively. Fifty-seven caregivers and 43 children provided questionnaire at both baseline and discharge.

We did not find significant differences in basic characteristics (i.e., age, sex, diagnosis, CGAS at admission and discharge, and length of stay) between complete responders and incomplete responders except for diagnosis--patients with OCD had a high collection rate; patients with PDD had a low collection rate. Families of children with ADHD also had a low collection rate.

## Results

### Participant Characteristics

During the study period, 126 patients were discharged from the unit and participated in this study. Thirty-two patients (25.4%) began the study period in the unit, whereas the others were admitted over the course of the study period.

Table 1 shows the participant characteristics. The mean age at admission was 12.8 years old, and 60% of participants were girls. Patients were 10.9 years old at the onset of their disorder on average (SD = 2.6). About 17% of patients suffered from obsessive-compulsive disorder (OCD), 14% had eating disorders (mostly anorexia nervosa), 13% had pervasive developmental disorders (PDD), 11% had anxiety disorders other than OCD, 9% had adjustment disorders, 8% had attention deficit hyper-activity disorder (ADHD), 6% were diagnosed with schizophrenia, 6% had mood disorders and the remaining 16% had other disorders (mostly neurotic disorders, such as somatoform, dissociative, or personality disorders).

**Table 1:** Characteristics of Patients Discharged from Child Psychiatric Unit (N = 126)

	Mean or No.	SD or %
Age at admission	12.8	1.9
Sex		
Male	50	39.7%
Female	76	60.3%
Diagnosis		
Obsessive-compulsive disorder	21	16.7%
Eating disorders	18	14.3%
Pervasive developmental disorders	16	12.7%
Attention deficit hyper-active disorder	10	7.9%
Schizophrenia	8	6.3%
Mood disorders	8	6.3%
Other disorders	45	35.7%
Cognitive level		
Normal function	101	80.2%
Borderline function	18	14.3%
Mental retardation	6	4.8%
Comorbid physical condition		
Present	30	23.8%
Past psychiatric inpatient		
None	92	73.0%
Once	21	16.7%
Twice or more	12	9.5%
Admission form		
Voluntary	67	53.2%
Compulsory	59	46.8%
Single or no parents	28	22.2%

Most children showed normal intellectual functioning. A comorbid physical condition was present in one-fourth of the participants. Frequently observed physical diseases included asthma, atopic disease, allergy, and epilepsy. More than one-third of the children exhibited current or past self-harming behaviors, and more than 30% had shown disruptive behavior. About two-thirds of the children had the experience of school refusal. Of the participants, 23% were victims of bullying. Nine children had been abused. This was the first admission to a psychiatric unit for 73% of the patient; approximately 47% of cases were compulsory admissions with the consent of parents. Mothers were the predominant primary caregivers; the mean age of parents was 45.3 years for fathers and 42.1 years for mothers. About 25% of participants were raised by single parents or were orphaned.

### Main aim of inpatient treatment

The main aim of inpatient treatment (multiple answers) was reduction of symptoms for 65.1% of the children, improvement of sociability and interpersonal relationship in 71.4%, adjustment of surrounding conditions for 42.9%, adjustment of pharmacotherapy in 39.7%, and close assessment for 12.7%.

### Treatment during stay

The treatments during patients' stays at the unit are summarized below. The mean length of inpatient stay was approximately eleven months (335.4 days, SD = 336.2) and ranged from 10 days to 5 years (median = 245 days). Individual psychotherapy sessions were offered to patients 1.39 times per week (SD = 0.92), family therapy 1.56 times per month (SD = 0.88), group psychotherapy 0.44 times per month (SD = 0.42), and occupational therapy 0.73 times per month (SD = 0.66). Forty-two children (33.3%) had to be segregated or restrained during some part of their stay. Seventy children (55.6%) attended the in-hospital school.

### Symptoms/Functioning at Baseline

Table 2 shows the means and standard deviations for each scale at baseline and discharge.

**Table 2:** Comparisons of Scales between Baseline and Discharge

	Baseline		Discharge				Effect size
	**Mean**	**SD**	**Mean**	**SD**	**Paired t**	**p**	**Cohen's d**

Psychiatrist (n = 126)							
CGAS	38.1	13.9	57.9	14.6	-14.38	0.00	1.39
Main Caregiver (n = 56)							
CBCL							
Full Score	49.9	30.5	38.7	26.6	3.8	0.00	0.39
Internalization	17.6	10.8	12.7	9.1	4.31	0.00	0.49
Externalization	11.5	11.7	9.4	9.1	2.08	0.04	0.21
Withdrawn	4.7	3.4	3.2	2.9	3.76	0.00	0.47
Somatic complaints	3.1	3.5	2.1	2.7	3.32	0.00	0.34
Anxious/depressed	10.4	6.1	7.8	5.2	3.61	0.00	0.46
Social problems	4.3	3.4	3.8	2.9	1.59	0.12	0.17
Thought problems	2.9	2.4	2.1	2.3	2.93	0.00	0.37
Attention problems	6.2	3.8	6.1	4.2	0.25	0.81	0.02
Delinquent behavior	2.8	3.6	2.1	2.4	1.85	0.07	0.21
Aggressive behavior	8.8	8.5	7.2	7.0	2.04	0.05	0.20
Child (n = 42)							
YSR							
Full Score	63.1	26.4	53.7	28.9	2.4	0.02	0.34
Internalization	23.7	11.6	18.4	11.2	3.07	0.00	0.46
Externalization	12.9	9.5	12.1	9.2	0.82	0.42	0.09
Withdrawn	5.9	3.3	4.4	2.7	3.12	0.00	0.49
Somatic complaints	4.2	4.3	2.8	3.1	2.44	0.02	0.38
Anxious/depressed	14.4	7.2	11.7	7.5	2.37	0.02	0.37
Social problems	6.5	3.3	5.4	3.1	2.21	0.03	0.31
Thought problems	3.3	3.0	2.8	2.8	1.13	0.26	0.18
Attention problems	8.1	3.7	7.5	3.4	0.98	0.33	0.17
Delinquent behavior	3.6	2.9	2.9	3.0	1.87	0.07	0.22

CGAS, Child Global Assessment Scale; CBCL, Child Behavior Checklist; YSR, Youth Self Report.

The mean CGAS score at admission was 38.1 (SD = 13.9). CBCL and YSR scores at baseline were high--most patients' syndrome scores were in the clinical or borderline range. Internalizing subscales were higher than externalizing subscales.

### Longitudinal Comparisons between Baseline and Discharge

Table 2 provides the results of the comparison between baseline and discharge for each scale. Psychiatrists' ratings showed that CGAS significantly improved at discharge (p < 0.01). Caregivers also reported that most of their children's problems improved during inpatient stay. YSR full score (p < 0.05) and internalizing behavior scores (p < 0.01) were significantly lower at discharge than at baseline.

### Factors Associated with Global Assessment Improvements

Between admission and discharge, psychiatrists' ratings showed a 19.8-point improvement in CGAS scores (SD = 15.5). Table 3 shows the associations between CGAS improvement and other factors. As expected, CGAS improvement was correlated with more serious symptoms at admission and with less serious symptoms at discharge. We observed differences among diagnostic groups; post-hoc comparisons showed greater improvement in children with OCD compared to children with schizophrenia. Patients showed better improvement when admission was involuntary.

**Table 3:** Factors Associated with Improvement in CGAS Score

	n	CGAS Change	Statistical value	p
Variables at Admission				
Age at admission	126		r = 0.10	0.27
Sex			t = 2.58	0.01
Male	50	24.1		
Female	76	17.0		
CGAS at admission	126		r = -0.51	0.00
Diagnosis^†^			F = 2.19	0.049
PDD	16	20.3		
ADHD	10	18.7		
Eating disorders	18	21.8		
OCD	21	26.5		
Schizophrenia	8	4.5		
Mood disorders	8	18.1		
Other disorders	45	18.9		
Admission mode			t = -2.94	0.00
Voluntary	67	16.0		
Involuntary	59	24.1		

Variables during the Treatment Process				
Group psychotherapy per month	125		r = 0.33	0.00
Restraint			t = 2.37	0.02
No experience of restraint	97	17.7		
Was restrained during stay	28	27.5		
In-hospital school			t = 3.71	0.00
Attended	71	23.9		
Not attended	53	14.1		
Length of stay	125		r = 0.25	0.00

Variables at Discharge				
Age at discharge	124		r = 0.23	0.01
CGAS at discharge	126		r = 0.58	0.00
Discharge			t = -6.96	0.00
Completed	105	22.8		
Interrupted	20	3.9		

†Post-hoc analysis revealed significant differences between OCD and Schizophrenia

Only key variables and variables that were p < 0.05 are presented.

CGAS, Child Global Assessment Scale; PDD, Pervasive developmental disorders; ADHD, Attention deficit hyper-active disorder; OCD, Obsessive compulsive disorder.

Among the treatment variables, frequency of group psychotherapy and attendance at the in-hospital school were significantly related to CGAS improvement. Patients who required restraining during the stay showed more positive changes in their CGAS score. Length of stay was also significantly related to improvement; those participants who stayed longer showed the greatest improvements. Older patients and those whose discharge was planned also showed significant improvement.

A linear regression clarified the effects that enhance psychological outcomes, using CGAS improvement as the dependent variable. Independent variables included sociodemographic factors (e.g., age, sex, diagnosis) and the factors at admission or during hospital stay associated with CGAS change. We did not enter the variables obtained at discharge because the aim of this analysis was to identify outcome predictors. Table 4 shows the results of the linear regression. Factors related to improvement include age, lower CGAS scores at admission, non-schizophrenia diagnosis, and group therapy frequency.

**Table 4:** Linear Regression with Change of CGAS as Dependent Variable

	Beta	p
Age at admission	0.30	0.00
Sex^†^	-0.16	0.053
CGAS at admission	-0.45	0.00
Diagnosis^‡^		
Pervasive developmental disorders	-0.04	0.62
Attention deficit hyper-activity disorder	-0.06	0.44
Eating disorders	0.13	0.12
Obsessive compulsive disorder	-0.02	0.79
Schizophrenia	-0.22	0.00
Mood disorders	-0.09	0.23
Group psychotherapy per month	0.25	0.00
Admission Mode^§^	0.07	0.45
Restrained^¶^	0.08	0.37
In-hospital school^††^	-0.03	0.74
Length of stay	0.12	0.12

Adjusted R2 = 0.50, p = 0.00

^† ^[Male = 0, Female = 1]

^‡ ^[Other disorders = 0]

^§^[Voluntary = 0, Involuntary = 1]

^¶^[Never = 0, Had restrained during stay = 1]

^††^[Not Attended = 0, Attended = 1]

CGAS, Child Global Assessment Scale.

## Discussion

This is the first empirical study to assess treatment effects at admission and discharge among inpatients of a child and adolescent psychiatric unit in Japan. This study clarifies which patient characteristics are most responsive to inpatient treatment using comprehensive questionnaires completed by the psychiatrist, the primary caregiver, and the child. This study also indicates the factors associated with improved outcomes.

### Participant Characteristics Admitted to the Inpatient Unit

Symptom severity and problematic behaviors were classified in the clinical range at baseline based on the questionnaire responses of all three groups (i.e., psychiatrist, caregiver, and participant).

As in previous studies, the diagnoses of children admitted to our unit varied. The most common diagnoses were OCD and eating disorders, followed by PDD [17,18].

The incidence of children who were orphaned or from single-parent families was high (22.2%), even after taking into account Japan's increasing divorce rate [19]. These data may suggest that children who do not live with both parents are more prone to psychiatric problems that require inpatient treatment, although we cannot establish a causal relationship from this data.

Nearly 27% of the children in this study had previously been admitted as psychiatric inpatients. These data suggest that there is a high recurrence rate in children with psychiatric problems, so follow up treatments after discharge are needed.

Patients' mean length of stay was 335 days (median = 245 days). Although there is currently a trend for short stays in child psychiatric wards, this stay is still relatively long compared to other countries. Long length of stay in adult psychiatric unit is also a significant problem in Japan. In adult setting, this due mainly to the lack of community mental health service and also the fee-for-service payment system which gives incentives to hospitals to keep the patients hospitalized. In child setting, in addition to the lack of community resources especially residential facilities, factors associated with their families are important. Many children with long lengths of stay have not only severe psychiatric problem but also tend to have family problem, such as abuse and poor upbringing ability. In such case, rather than discharge children to their family, we try to find another setting, which is usually difficult. The development of community treatments such as residential facilities and outreach services are necessary to help solve this problem of extended in-patient treatment regimes.

### Outcomes of Youth Psychiatric Unit Treatment

As measured by CGAS at admission, patients showed major functioning impairment in several areas. At discharge, CGAS scores were 20 points higher on average, in the range of 'Variable functioning with sporadic difficulties or symptoms in several but not all social areas'. Although these results may be biased considering that the attending psychiatrist made the CGAS ratings, certain children's global functioning seemed to improve during inpatient treatment: Children rated their own behavior and functioning (using the YSR) as having significantly improved. Caregivers also agreed that most of their children's symptoms had improved during the stay. These results are consistent with findings reported by Gavidia-Payne et al., in the Australian setting with shorter length of stay [4]. Future study in Japan should measure the improvement of the child not only at discharge but during the inpatient treatment to know when the change occurs; if this is known, it may contribute to earlier discharge.

### Factors associated with better outcomes

A linear regression analysis revealed that older age and lower CGAS scores at admission, as well as the type of diagnosis and frequent group therapy sessions were associated with greater improvement as measured by CGAS. In short term inpatient unit, Mathai and Bourne have found no meaningful conclusions as to what sort of patient would benefit most from an admission to the unit [20]. So this result may suggest that some patient might benefit from longer term hospitalization, but since our study used only CGAS for measuring improvement, this result should be interpreted cautiously, and future researches using broad band measures are needed.

The result that the older youths showed better outcomes than the younger youths be counter-intuitive; however, most of our participants were less than 15 years old, which is still very young. Age may have much to do with intellectual or developmental problems, which are difficult to overcome rapidly (e.g., children with mental retardation or other developmental disorders were significantly younger than those with normal IQs). Because the standard deviation of age was only 1.9 years, however, future studies should include more age groups.

Patients with schizophrenia made poor progress, whereas patients with obsessive-compulsive disorder showed improvement. Children with severe positive symptoms of schizophrenia, such as hallucination and delusions, are not usually admitted to our unit and instead are sent to a closed adult unit. Thus, patients with positive symptoms are not common, which makes improvement difficult. If the study had been conducted for all of the inpatients with schizophrenia, including those with severe positive symptoms, our result may have differed; therefore, making conclusions regarding the effect of inpatient treatment on schizophrenia is difficult based on this study. Studies that focus on specific diagnostic groups and disease-specific scales are needed.

Apart from group psychotherapy, treatment variables showed no relationship with changes in CGAS scores. This may have been due to the fact that the treatment offered to children in this unit does not vary greatly.

### Limitations

Several limitations apply to this study. First, we lacked a comparison or control group, which is a common problem for this type of study. The severity of patients' disorders and their urgent need for hospitalization made establishing a comparison group both practically and ethically difficult. To avoid this difficulty, a future study should compare different types of treatment within an inpatient unit. Second, although basic data and psychiatrist ratings were obtained for all participants, the response rate of caregivers and children was not high. Thus, self-selection bias is a potential problem. Other studies have also suffered from this problem, and the difficulties of conducting evaluation research in an inpatient unit have been described elsewhere [4,5]. Third, we did not assess patients' long-term outcomes in this study. We have planned a follow-up of these participants after discharge. Finally, we obtained data from one hospital. Although this hospital is one of the few that teach child and adolescent psychiatry in Japan and many other hospitals follow similar treatment methods, generalization of our results to other hospital is unknown. Thus, a multicenter study is needed. Fourth, although families and children have rated outcome using CBCL and YSR, CGAS was the only clinician rated outcome measure used in this study. This due to the lack of broad band measure such as HoNOSCA in Japan, but future studies should include such scales.

## Conclusion

This prospective evaluation in a child and adolescent inpatient unit demonstrated patients' clinical improvement. No prior study has evaluated the outcomes of inpatient treatment for children with psychiatric problems in Japan. This study supports the further development of the medical infrastructure needed for youth with psychiatric disorder. In addition, this study provides ways of identifying those who will benefit most from inpatient treatment. The factors associated with improved functioning included age, lower functioning at admission, the frequency of group therapy during hospitalization, and the type of psychiatric diagnosis.

## Competing interests

The authors declare that they have no competing interests.

## Authors' contributions

YS, KS, and MK have made substantive contributions to the conception of the study; KW, MK, and MU have contributed to the study implementation. All authors read and approved the final manuscript.
